# Establishment and biological characteristics of fibroblast cell lines obtained from wild corsac fox

**DOI:** 10.1007/s11626-020-00527-5

**Published:** 2020-11-12

**Authors:** Xihe Li, Yunxia Li, Xiaojie Yan, Xiaonan Guo, Yongli Song, Baojiang Wu, Siqin Bao, Guifang Cao, Jitong Guo, Qingyuan Sun

**Affiliations:** 1 Research Center for Animal Genetic Resources of Mongolia Plateau, College of Life Sciences, Inner Mongolia University, Hohhot, 010070 China; 2 Inner Mongolia Saikexing Institute of Breeding and Reproductive Biotechnology in Domestic Animal, Hohhot, 011517 People’s Republic of China; 3 College of Veterinary Science, Inner Mongolia Agricultural University, Hohhot, 010018 People’s Republic of China; 4 Institute of Zoology, Chinese Academy of Science, Beijing, 100101 People’s Republic of China

## Introduction

Animal genetic resources are basic materials for life science studies and are important resources for the survival and economic development of humanity (Min 2010; Liu 2011; Cheng *et al*. 2018). Wild animal resources are important components of China’s natural resources (Qu 2018). The low-temperature preservation of animal cells is an effective method for the protection of animal genetic resources and is particularly important in the conservation of endangered animal species (Shang 2018). Isolation and cultivation of fibroblasts from different animal tissues for the establishment of fibroblast cell lines are commonly used methods for the preservation of live tissue genetic materials. These cell materials can be stored for “half-permanent” in a – 196°C liquid nitrogen environment (Daorna *et al*. 2013). Preserved animal genetic resources can be used for animal cloning to revive corresponding species and provide materials for experiments in the fields of stem cells, genetic engineering, cell engineering, and molecular biology (Min 2010).

Corsac fox (*Vulpes corsac*) is mainly lhabitated in Central Asia and is the smallest species among foxes (Zhao *et al*. 2016b) (Zhao *et al*. 2016a, b). Previous studies on corsac were mainly focused on their genetics and systematic taxonomy (Graphodatsky *et al*. 2008; Zhao et al. 2016a; Shang *et al*. 2017), biochemistry, and physiology (I V *et al*. 1900; Pozio *et al*. 1992; Tang et al. 2001; Kuzmin *et al*. 2004; Botvinkin et al. 2008; Odontsetseg et al. 2009; Ito *et al*. 2013), as well as ecological distribution (Mal'kova 2000; Tang *et al*. 2004). Presently, there is no report available on the establishment of fibroblast cell lines in corsac fox and its biological characteristics.

## Results and discussion

**Fig 1:**
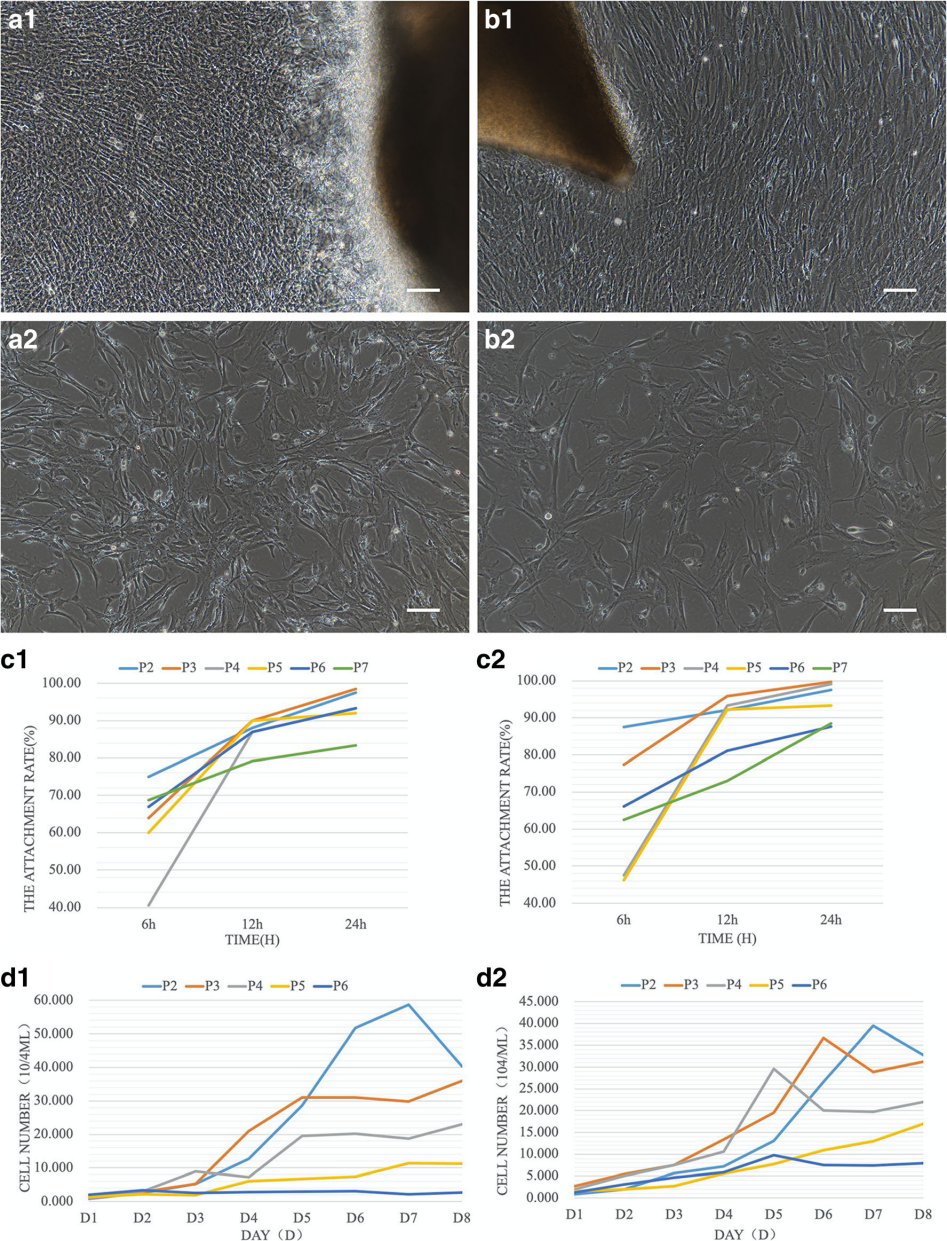
Culture and establishment of primary fibroblasts from corsac fox. (*a1*–*a2*) Character of trachea fibroblasts in P0, P7. (*b1*–*b2*) Character of cartilage fibroblasts in P0, P7; *Bar* = 100 μm. (*c1*) Growth curve of trachea fibroblasts from p2–p7 for 0–24 h. (*c2*) Growth curve of cartilage fibroblasts from p2–p7 for 0–24 h. (*d1*) Growth curve of trachea fibroblasts from p2–p6 for D1–D8. (*d2*) Growth curve of cartilage fibroblasts from p2–p6 for d1–d8.

One female corsac fox from Horinger County of Inner Mongolia was used to obtain the required tissue samples. The prepared tissue samples were cut into 0.1–0.5 mm^3^ tissue blocks and were placed at the bottom of T25 culture flasks (Corning, Shanghai, China). Six- to 8-mL culture medium (MEM-Alpha containing 10% FBS and 1% P/S) (Gibco, Shanghai, China) was slowly added to each culture flask and cultivated in an incubator with 38°C and 5% CO_2_ for 6–8 h to obtain primary cell line culture (Li *et al*. 2013). When cells reached 80% confluency, the cell culture medium was discarded, and the cells were gently rinsed by 2 mL of DPBS(Gibco). Subsequently, 1 mL of 0.25% trypsin(Gibco) was added, and the cells were digested for 3 min, and then 2 mL of culture medium (MEM-Alpha containing 10% FBS and 1% P/S) was added to terminate the digestion. Cells were collected before centrifugation at 1500 r/min for 5 min and seeded at a density of 10^5^/mL in 6-well plates (Corning, Shanghai, China) and were cultivated continuously in an incubator at 38°C and 5% CO_2_ (Wang 2011). Observations indicated that 13 and 11 days are required to establish a primary fibroblast cell line from corsac tracheal and cartilage tissues, respectively (Fig. 1*a1* and *b1*). At passages P0–P3, the two types of fibroblast cells appeared highly three dimensional. As culture duration and passage number increased to P4–P7, cells gradually become flat (Fig. 1*a2* and *b2*).

Viability of fibroblast cells was measured before and after cryopreservation. According to the instructions of the manufacturer (Mu 2018), 10 μL trypan blue (Gibco) was added to 40 μL cell suspension, and incubation took place for 5 min. After the staining was completed, the cell color was observed. Transparent cells were regarded as viable cells, while pale blue cells were dead cells. The viability of P3–P7 tracheal fibroblast cells before and after cryopreservation ranged from 91.30 to 96.30% and from 61.10 to 80.00%, respectively. The viability of P3–P7 cartilage fibroblast cells before and after cryopreservation ranged from 90.53 to 98.08% and from 76.67 to 90.20%, respectively. The viability of fibroblast cells obtained from the two different tissues after cryopreservation and thawing was significantly decreased in comparison with the viability rates obtained before cryopreservation, and also the cell viability was also decreased following the number of passages increased. The adherence rate was used to determine the growth and proliferation status of the cells (Mu 2018). The adherence rate of the two types of fibroblast cells significantly increased from 60% at around 6 h of cultivation to more than 90% after 12 h of cultivation, which was maintained after 24 h. The statistical results for the adherence rate of the two types of fibroblast cells show that tracheal fibroblast cells proliferated faster than cartilage fibroblast cells (Fig. 1*c1* and *c2*).

The growth curve is an important parameter for the measurement of the cell viability as well as other biological characteristics (Blackburn *et al*. 1998). 2.4 × 10^5^ cells were seeded at a density of 1 × 10^4^ cells per well in a 24-well plate (Corning). Three wells constituted one group, and 8 groups were placed in the incubator for cell cultivation. This procedure was carried out for 8 consecutive d. A cell growth curve was plotted using the number of days of culture as the *x*-axis and the daily cell count as the *y*-axis. The calculated results were used to plot a line chart of changes in adherence rate at different time points. Growth curve results showed that the proliferative capacity of tracheal fibroblasts was faster than that of the cartilage. On days 4–6 after seeding, the two types of fibroblast cells entered the logarithmic growth phase. In P2-P6, as the number of passages increased, the growth rate of cells decreases (Fig. 1*d1* and *d2*).

**Fig 2:**
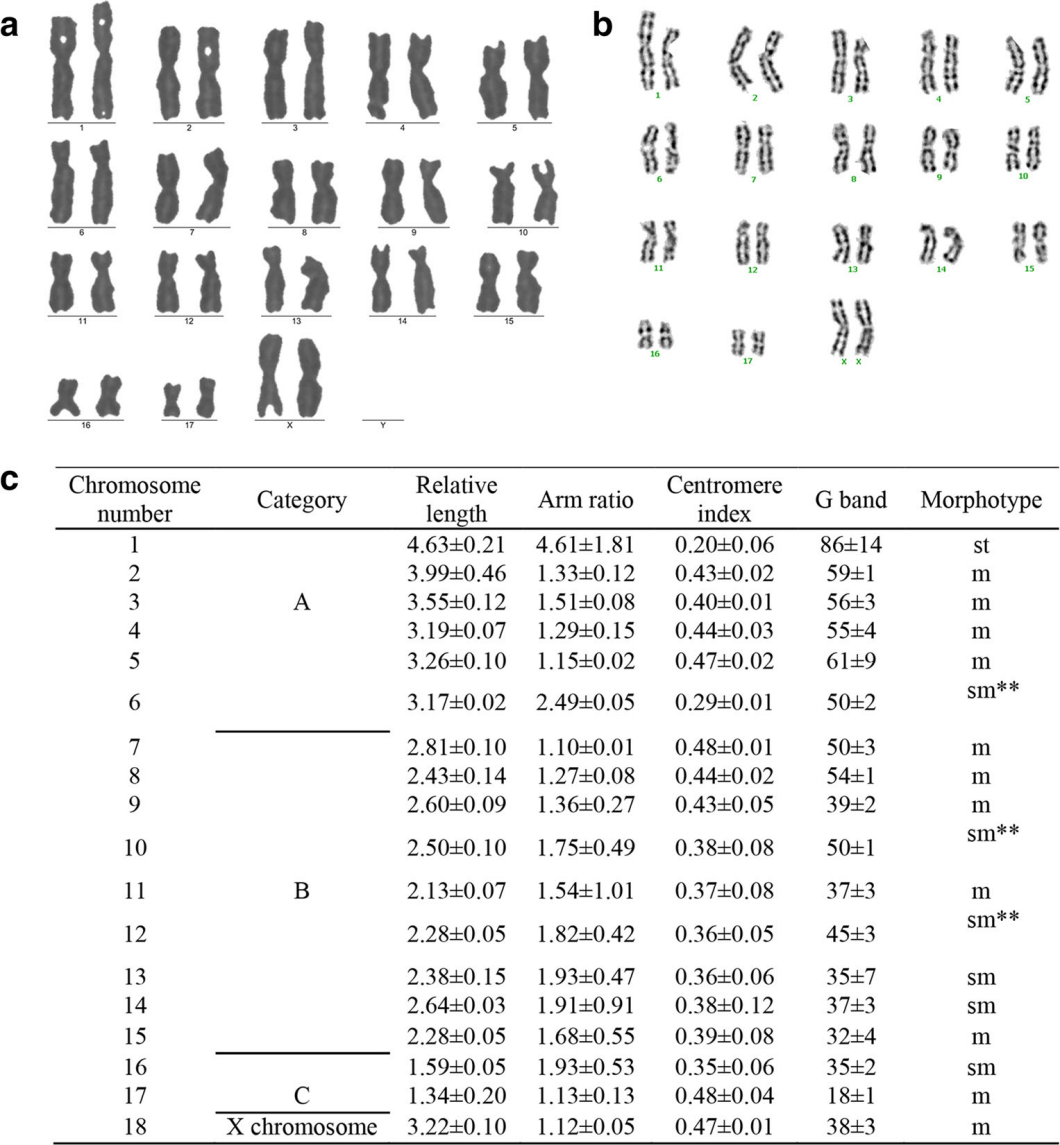
Karyotype and G-band analysis of chromosome in corsac fox. (*a*) Metaphase of chromosome in corsac fox(2n = 36, XX), the *left* is metaphase, the *right* is the karyotype arrangement. (*b*) G-band of chromosome in corsac fox (17 chromosomes were autosomal. The chromosome morphology was 1st + 10 m + 6sm, another was sex chromosomes XX, which had a morphology of m). (*c*) Statistics analysis of chromosome karyotype and G-banding in corsac fox. *A.* Maximum group, *B.* medium group; *C. minimum* group; SM, Submetacentric chromosome; M, central kinetochore chromosome; ST, acrocentric chromosome.

Chromosome karyotype and G-banding were analyzed. The analysis and comparison of the arrangement and the number of chromosomes were conducted using a banding technique based on chromosome length, centromere position, the ratio of long and short arms, and the presence/absence of satellite chromosomes (Mu 2018). Manual interpretation combined with automatic sorting using a cytogenetic workstation was applied for chromosome karyotyping. Cooled chromosome slides were added to pre-heated 0.0125% trypsin and incubated in a 37°C water bath for 40–50 s for digestion and followed by 10–15 min of Giemsa staining. After the slides were rinsed and dried, the cytogenetic workstation was used for photography and G-banding analysis. P4 trachea-derived fibroblast cells with stable passage were used to prepare chromosome samples. Fifty cell samples showing well-separated chromosomes with a high division index were observed. Statistical results show that the number of chromosomes in Corsac fox fibroblasts obtained from this study was 2n = 36 (Fig. 2*a* and *b*), among which 17 chromosomes were autosomal, with a morphology of 1st + 10 m + 6sm, and the pair of sex chromosomes were XX with a morphology of m (Fig. 2*c*). In this study, among the 50 dividing cells with metaphase chromosomes, 42 dividing chromosomes were identified with normal diploid characteristics, showing that the chromosome phase of the established fibroblast cell line is stable.

**Fig 3:**
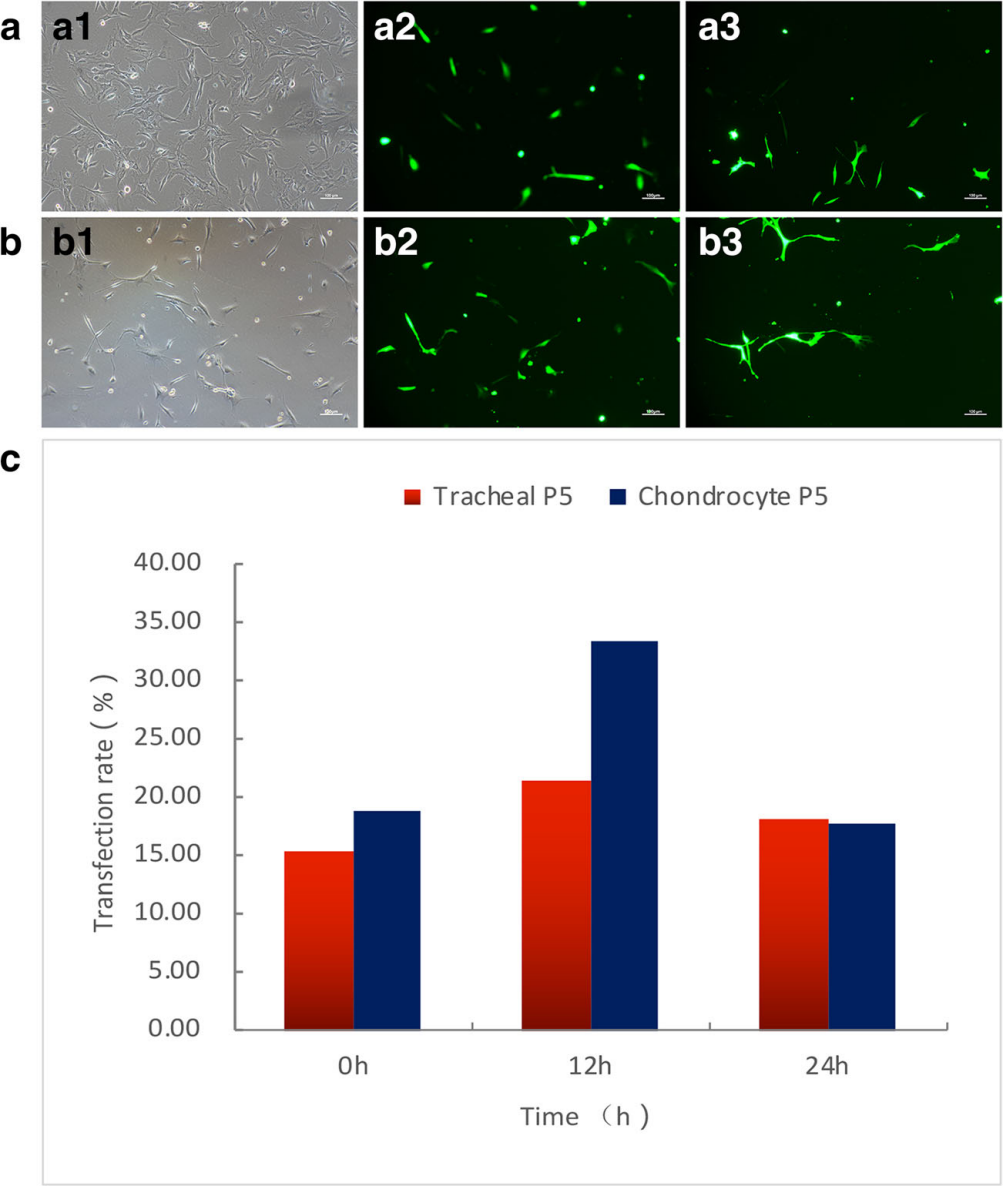
Fibroblast fluorescent protein expression transfected plasmid in corsac fox. (*a1*–*a3*) Fluorescent protein expression transfected plasmid in tracheal fibroblasts at 0 h, 12 h, and 24 h after transfection. (*b1*–*b3*) Fluorescent protein expression transfected plasmid in chondrocyte fibroblasts at 0 h, 12 h, and 24 h after transfection. *c* Comparison of transfection rate of in corsac fox two fibroblasts at 0 h, 12 h, and 24 h after transfection (more than 20%).

Transfection, the process of introducing target genes into cells, was used as a technique for studying gene function and genetic stability (Zhang *et al*. 2017). Two types of cartilage and tracheal fibroblast cells were used in this experiment, and the transfection reagent Lipofectamine™ 2000 (Invitrogen, Shanghai, China) and cells were mixed for transfection according to the instruction of the manufacturer. Six hours after transfection, the expression status of green fluorescent protein was examined under 488 nm green laser before cells were cultured for 12–24 h in a CO_2_ incubator. We selected five different views at the corresponding time points for photography and calculated the associated transfection rate. Results of the experiment show that the transfection rates of cartilage and tracheal fibroblast cells reached the highest at 12 h of transfection, with 35% and 20% cells transfected (Fig. 3*a*, *b*, and *c*), respectively.

## Conclusions

Corsac fox used in this study was obtained from Horinger County in Inner Mongolia. The tracheas and cartilage tissues were collected and used to establish of primary fibroblast cell lines. The morphology, growth rate, adherence rate, cryopreservation viability, karyotype, and G-banding of fibroblasts derived from two types of tissues were investigated, and the liposomal transfection rate was used to evaluate the genetic stability of the fibroblasts. This is the first report for establishment and biological characteristics of the fibroblast cell lines obtained from wild corsac fox.

## References

[CR1] Blackburn H, Lebbie SHB, van der Zijpp AJ (1998). Animal genetic resources and sustainable development. Animal genetic resources and sustainable development, 6WCGALP/FAO symposium.

[CR2] Botvinkin AD, Otgonbaatar D, Tsoodol S, Kuzmin IV (2008). Rabies in the Mongolian steppes. Dev Biol (Basel).

[CR3] Cheng P, Lu F, Zhang P, Ma J, Wang R (2018). Current situation and development thinking on protection and utilization of biological Germplasm resources in China. China Sci Technol Resour Rev.

[CR4] Daorna LG, Wang R, Li Y, Dai Y, Li X, Li Y, Li Y (2013). Biological characteristic analysis on different Lynx tissue cells cultured in vitro. China Anim Husb Vet Med.

[CR5] Graphodatsky AS, Perelman PL, Sokolovskaya NV, Beklemisheva VR, Serdukova NA, Dobigny G, O'Brien SJ, Ferguson-Smith MA, Yang F (2008). Phylogenomics of the dog and fox family (Canidae, Carnivora) revealed by chromosome painting. Chromosom Res.

[CR6] I V K m, GN S, AD B, EI R (1900). Epizootic situation and prospectives of rabies control among wild animals in the south of Western Siberia. Zh Mikrobiol Epidemiol Immunobiol.

[CR7] Ito A, Chuluunbaatar G, Yanagida T, Davaasuren A, Sumiya B, Asakawa M, Ki T, Nakaya K, Davaajav A, Dorjsuren T, Nakao M, Sako Y (2013). Echinococcus species from red foxes, corsac foxes, and wolves in Mongolia. Parasitology.

[CR8] Kuzmin IV, Botvinkin AD, McElhinney LM, Smith JS, Orciari LA, Hughes GJ, Fooks AR, Rupprecht CE (2004). Molecular epidemiology of terrestrial rabies in the former Soviet Union. J Wildl Dis.

[CR9] Li Y, Fang Y, Song D, Ma H, Hou B, Li Y (2013). Isolation and cultivation of cells from the tissue of trachea of rough-legged buzzard. J Nanchang Univ Nat Sci.

[CR10] Liu, Y. (2011). Legal system of protection of animal genetic resources in China [master, Lanzhou University]

[CR11] Mal'kova MG (2000). Ecological and epizootic characteristics of various zonal types of natural reservoirs of alveococcosis in the Omsk region. Med Parazitol.

[CR12] Min, Y. (2010). Establishment and biological characteristics of fibroblast cell lines from three kinds of tissues of Tibetan mastiff [master, northwest Minzu University]

[CR13] Mu, Y. (2018). Establishment of fibroblast cell linesin vitro culture system from Chinese Zocor(Zokor) and its biological characteristics analysis [master, Inner Mongolia University]

[CR14] Odontsetseg N, Uuganbayar D, Tserendorj S, Adiyasuren Z (2009). Animal and human rabies in Mongolia. Rev Sci Tech.

[CR15] Pozio E, Shaikenov B, La Rosa G, Obendorf DL (1992). Allozymic and biological characters of Trichinella pseudospiralis isolates from free-ranging animals. J Parasitol.

[CR16] Qu, H. (2018). The present situation and Prospect of endangered wildlife protection in China. *The Farmers Consultant*(8)

[CR17] Shang, H. (2018). Establishment and characterization of ear skin fibroblast cell lines derived from Wandong bulls [master, Anhui Agricultural University]

[CR18] Shang S, Wu X, Chen J, Zhang H, Zhong H, Wei Q, Yan J, Li H, Liu G, Sha W, Zhang H (2017). The repertoire of bitter taste receptor genes in canids. Amino Acids.

[CR19] Tang C, Chen J, Tang L, Cui G, Qian Y, Kang Y, Lv H (2001) Comparison observation on the mature alveolar of Echinococcus sibiricensis and Echinococcus multilocularis in the experimentally infected white mice. Acta Biol Exp Sin (04):261–268 12549203

[CR20] Tang CT, Quian YC, Kang YM, Cui GW, Lu HC, Shu LM, Wang YH, Tang L (2004). Study on the ecological distribution of alveolar Echinococcus in Hulunbeier Pasture of Inner Mongolia,China. Parasitology.

[CR21] Wang, W. (2011). Primary Schwannoma cell culture and personalized study of NF2 gene mutation in neurofibromatosis type ǁ [master, Tianjin Medical University]

[CR22] Zhang Q, Guo X, Gao P, Jin Y, Li M, Cheng Z, Zhang N, Yue B, Liu H, Cao G, Li B (2017). In vitro culture and identification of ear marginal fibroblast cell lines derived from Mashen pigs. China Anim Husb Vet Med.

[CR23] Zhao C, Zhang H, Liu G, Yang X, Zhang J (2016). The complete mitochondrial genome of the Tibetan fox (Vulpes ferrilata) and implications for the phylogeny of Canidae. C R Biol.

[CR24] Zhao C, Zhang J, Zhang H, Yang X, Chen L, Sha W, Liu G (2016). The complete mitochondrial genome sequence of the corsac fox (Vulpes corsac). Mitochondrial DNA A.

